# The role of IgE specific for galactose-α-1,3-galactose in predicting cetuximab induced hypersensitivity reaction: a systematic review and a diagnostic meta-analysis

**DOI:** 10.1038/s41598-020-78497-7

**Published:** 2020-12-07

**Authors:** Cristian Virgil Lungulescu, Bogdan Silviu Ungureanu, Adina Turcu-Stiolica, Valentina Ghimpau, Stefan Alexandru Artene, Irina Mihaela Cazacu, Alexandru Florian Grecu, Venera Cristina Dinescu, Adina Croitoru, Simona Ruxandra Volovat

**Affiliations:** 1 Department of Oncology, University of Medicine and Pharmacy of Craiova, Craiova, Romania; 2 Research Center of Gastroenterology and Hepatology, University of Medicine and Pharmacy of Craiova, Craiova, Romania; 3 Department of Pharmacoeconomics, University of Medicine and Pharmacy of Craiova, Craiova, Romania; 4 Pharmacy of Craiova Doctoral School, University of Medicine, Craiova, Romania; 5 Department Biochemistry, University of Medicine and Pharmacy of Craiova, Craiova, Romania; 6 Fundeni Clinical Institute, Titu Maiorescu University, Bucharest, Romania; 7 Department of Orthopedics, University of Medicine & Pharmacy of Craiova, Craiova, Romania; 8 Health Promotion and Occupational Medicine Department, University of Medicine & Pharmacy of Craiova, Craiova, Romania; 9 Department of Oncology, University of Medicine and Pharmacy “Grigore T Popa”, Iasi, Romania

**Keywords:** Cancer, Oncology

## Abstract

Recombinant monoclonal antibodies are used for treating various diseases, from asthma, rheumatoid arthritis, and inflammatory bowel disease to cancer. Although monoclonal antibodies are known to have fewer toxic reactions compared with the conventional cytotoxic antineoplastic drugs, the cases of severe systemic hypersensitivity reaction (HSR) should be acknowledged. Our aim was to assess the diagnostic accuracy of the anti-IgE for galactose-α-1,3-galactose in patients with HSRs to cetuximab. We searched in PubMed, Cochrane Library, Scopus, and World of Science databases to July 1st, 2020. We included a total of 6 studies, with 1074 patients. Meta-analysis was performed using bivariate analysis and the random-effect model. The pooled sensitivity was 73% (95% CI 62–81%) and the pooled specificity was 88% (95% CI 79–94%). We had not found significant heterogeneity and, despite some discrepancies in the nature of data available in the analysed studies, we draw the conclusion that the presence of cetuximab specific IgE (anti cetuximab antibody) and/or galactose-α-1,3-galactose shows moderate to high sensitivity and specificity of developing an HSR. More studies are needed to establish a protocol necessary for the proper prediction and avoidance of HSR related to cetuximab.

## Introduction

Recombinant monoclonal antibodies are currently used in the treatment of various diseases, from asthma, rheumatoid arthritis and inflammatory bowel disease to cancer^1,2^. Bevacizumab, panitumumab, trastuzumab, rituximab, cetuximab represent a new generation of molecules that are being used to treat different types of malignancies^3–6^. Although monoclonal antibodies are known to have fewer toxic reactions compared to the conventional cytotoxic antineoplastic drugs, several cases of severe systemic hypersensitivity reaction (HSR) have been reported^4,7–10^.

Cetuximab is a chimeric mouse–human immunoglobulin G1 monoclonal antibody against the epidermal growth factor receptor (EGFR) approved alongside chemotherapy, for KRAS wild-type colorectal cancer treatment, as well as for metastatic or loco-regionally advanced squamous-cell carcinoma of the head and neck^8,11–14^. This monoclonal antibody has a favourable safety profile, with but one exception: the life threatening hypersensitivity severe reaction (HSR)^15–19^.

According to the product’s label^20^, cetuximab can cause HSR CTCAE (Common Terminology Criteria for Adverse Events) Grades 3 and 4 in approximately 2–5% of the treated patients. Some reports discuss an increased incidence of hypersensitivity reactions that reaches 22% in some parts of the U.S.^16,21–23^, data sustained by the post-marketing pharmacovigilance reports. Clinical symptoms associated with this kind of reactions are the rapid onset of airway obstruction (laryngeal oedema, bronchospasm), hypotension, shock, unconsciousness, and myocardial infarction^24^. Serious infusion reactions, some fatal, occurred in approximately 3% of patients; cardiopulmonary arrest and/or sudden death occurred in 2% of patients receiving Erbitux in combination with radiation therapy^25^.

These severe reactions are known to develop within one hour after the first infusion^26^, but there were cases when the anaphylaxis reaction took place after several hours, and even after subsequent infusions^22^.

Different criteria that could predict the risk of an infusion reaction have been reported as follows: patients’ sex and race, smoking status, primary site of the tumour, allergy history, and whether antihistaminic premedication helped prevent this kind of severe adverse events^27,28^.

According to recent data, a type I allergic reaction might be involved, mediated by pre-existing IgE antibodies cross-reactive with the cetuximab molecule^15,29,30^. Studies have shown that the antibodies are specific for galactose-α-1,3-galactose^31^ (alpha-gal), an oligosaccharide which is present on both Fab portions of the cetuximab’s heavy chain. Alpha-gal is the only known critical epitope that reacts with the preformed IgE antibodies.

To our knowledge, a meta-analysis that assessed the diagnostic properties of alpha-gal in the diagnosis of HSR was never conducted. Therefore, our aim was to perform a diagnostic meta-analysis for finding out how accurate the antiIgE for alpha-gal is for diagnosis of cetuximab-induced HSRs.

## Methods

### Literature search strategy

We performed this meta-analysis according to the PRISMA for Diagnostic Test Accuracy (Preferred Reporting Items for Systematic Review and Meta-Analysis)^32^. We searched the literature published before July 1st, 2020, in PubMed, Cochrane Library, Scopus and World of Science. The following search terms were used: “HSR cetuximab”, “infusion reaction cetuximab”, “allergy cetuximab”, “galactose-α-1,3,-galactose”, “monoclonal antibodies reactions”. We did not set any restriction on study design, year of publication, study location or publication status. We took into consideration the retrieved articles’ references while trying to identify other potentially eligible publications.

### Inclusion and exclusion criteria

The following criteria were met by the studies that are included in the review: (1) studies including adult patients that were treated with cetuximab and had their adverse reaction evaluated; (2) studies that have tested their patients for the IgE antibodies specific for the carbohydrate alpha-gal; (3) data on true-positive (TP), true-negative (TN), false-positive (FP), false-negative (FN) were reported or could be calculated from the article; (4) studies written in English. We excluded case reports or case series, reviews, letters, studies reported only as meeting abstracts, case–control studies using healthy controls (high risk of bias). We also excluded studies where the relevant data were inaccessible or unclear.

### Data extraction and quality assessment

Two review authors (CVL, VG) independently performed the data extraction, in accordance with the inclusion and exclusion criteria listed above. Disagreements were resolved by discussion with a third author (BSU). For each study, the following data were recorded: name of the first author, year of publication, study design, site of malignancy, HSR reaction grade, correlations between IgE and HSR and summary of findings. The control population comprises patients treated with Cetuximab with no HSR. We extracted the values of true positives (TP), true negatives (TN), false positives (FP), and false negatives (FN) to calculate sensitivity and specificity.

We assessed the quality of the evidence using GRADE (Grading of Recommendations, Assessment, Development and Evaluation) Working Group criteria: risk of bias, consistency of effect, imprecision, indirectness and publication bias. We have assessed the risk of bias using QUADAS-2. Bias was assessed in the domains: participant selection, index test, reference standard, and flow/timing; and applicability was assessed in the first three domains only (participant selection, index test, and reference standard). Bias was independently graded as low, high, or unclear quality by two review authors (SAA, IMC). Discrepancies were resolved being moderated by a third review author (SRV).

### Statistical analysis

Individual study data were presented graphically as forest plots and summary receiver operating characteristic (sROC) curves that integrated ROC curves of primary studies. The bivariate random-effects model for meta-analysis of the pairs of sensibility and specificity was used. The statistical analysis was completed using Review Manager 5 (RevMan 5, The Nordic Cochrane Centre, Copenhagen, Denmark) and R-package mada. Descriptive statistics included the pooled sensitivity and pooled specificity of the studies. Area under the ROC curve was reported. An AUC of 0.5 represents an uninformative test and an AUC of 1 a test with 100% sensitivity and 100% specificity. The heterogeneity of the studies was assessed through the Higgin’s I^2^ (0% indicates no observed heterogeneity, and larger values show increasing heterogeneity) and χ^2^ test^33^. P-values for the difference in sensitivity and for the difference in specificity were reported. The significance level was 0.05.

## Results

**Figure 1:**
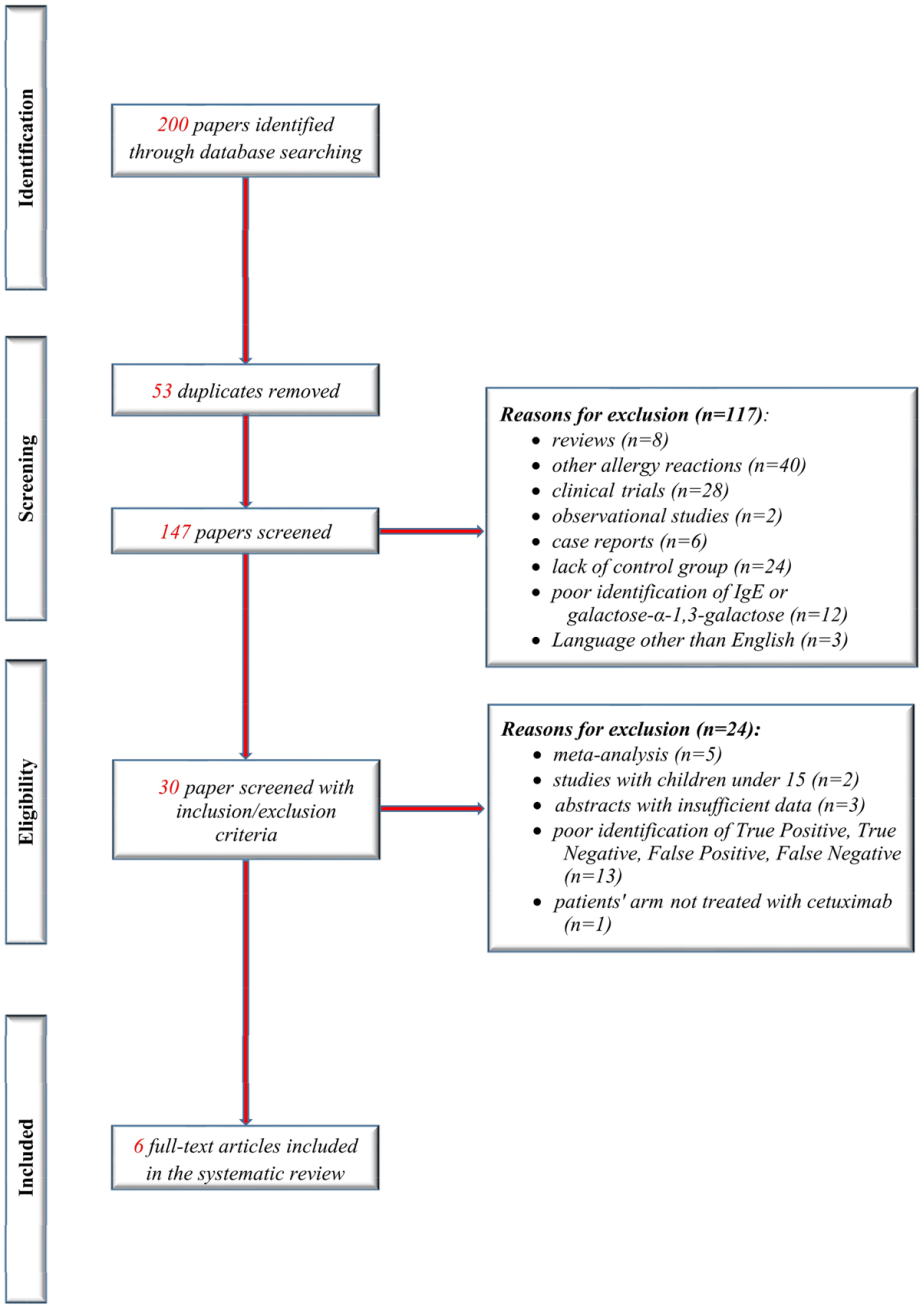
The flowchart of the studies included in this meta-analysis.

We have identified 6 articles that assessed the possibility of predicting anaphylaxis by using the alpha-gal, in response to cetuximab treatment. Details of the studies that met our inclusion criteria are presented in Table 1 with summary of findings as in GRADE. Figure 1 shows the flow diagram of the selection studies in the review, according to the PRISMA study selection process.

**Table 1:** Characteristics of the 6 included studies.

References	Study population	Study design	Site of malignancy	Cetuximab HSR	Correlation between IGE and HSR	Summary of findings
Chung^27^	n = 538	Retrospective analysis of cetuximab induced HSR and IgE specific for alpha-gal. Four groups of patients: group 1:76 patients who received treatment with cetuximab. Group 2,3,4 consisted of 462 patients: 72 healthy volunteers, 49 with history of head and neck cancer, 341 female control subjects. Method: ImmunoCAP	Colorectal cancer Head and neck Lung	26 HSR rated by the investigators: 13 low-grade, 12 high grade, 1 late response	Out of the 25 patients that had HSR, 17 had a positive test for IgE antibodies; 1 of the 51 subjects who did not have a hypersensitivity reaction had such antibodies before treatment with cetuximab	Specific antibodies for galactose-α-1,3-galactose were present in serum in most patients who had HSR to cetuximab
Dupont^26^	n = 229 (108 assessed for IgE antibody)	Retrospective study of a cohort of patients treated with cetuximab. Method: ELISA	Colorectal Head and neck Other	6 grade 1 reactions, 7 grade 2, 9 grade 3, 2 grade 4–5 reactions	The assay was positive in 13 out of 17 patients with HSR and in 17 out of 91 without HSR	Assessing the specific IgE values could be a valuable test to identify the high risk patients for developing HSR, but it needs further confirmation in prospective trials
Dupont^29^	n = 247	Multicenter, prospective cohort study. Method: ELISA	Head and neck Colorectal	12 patients experienced a HSR, of which 8 patients had a severe reaction (5 grade 3, and 3 grade 4 reaction)	33 patients out of 239 who did not experience severe HSR had high specific IgE values. 5 patients out of 8 who experienced severe HSR had high specific IgE values	Detection of pretreatment specific IgE is helpful in identifying patients with high risk of developing cetuximab-induced HSR
Iwamoto^15^	n = 12	History of cetuximab- induced HR within the past 3 months or initiation of cetuximab therapy within the next week. Method: ELISA	Head and neck cancer	2 patients with a grade 1 reactions; 2 with grade 3 reaction	6 patients were tested positive for IgE specific out of which 4 developed HSR. 6 patients were negative for IgE specific, none with HSR	The study concludes that evaluating drug-IgE Interaction it is an inviting method to identify high-risk patients for HSR
Maier^22^	n = 545	Retrospective case–control analysis of serum or plasma samples. Samples were obtained before administration of cetuximab. Method: ImmunoCAP	Colorectal Head and neck Ovarian cancer Pancreatic cancer NSCLC	21 patients developed SIR	Out of the 21 patients who experienced HSR 15 were IgE positive and 6 IgE negative. Out of the 524 without HSR 23 were IgE positive and 501 IgE negative	The study concludes that is unclear whether the plain presence of specific IgE predicts the risk to suffer a severe HSR upon administration or if a certain threshold is required
Mariotte^34^	n = 92	Retrospective study. Patients treated with cetuximab at François Baclesse Centre, Caen, France for which pre-treatment samples were available. Method: ELISA	Head and neck Colorectal Other	14 had HSR after first cetuximab administration. 6 had low to moderate reaction (grade 1–2). 6 had severe reactions (grade 3). 2 died following the HSR event (grade 4)	Anti-cetuximab IgE were considered positive in 14 out of 72 patients without HSR and in 10 out of 14 with HSR. In the 14 patients with HSR reaction, anti-cetuximab IgE levels reached a median level more than ten times bigger than in those without reaction	Pre-existing anti-cetuximab IgEs correlates with high risk for developing HSR at drug administration. The ELISA test presented in this study can predict a higher risk for developing HSR but not a reaction. There is no correlation between the levels of IgE and the severity of the reaction

The studies provided data for 1074 patients (Table 1). All of them included patients with head and neck cancer, and five of the studies included patients with colorectal cancer.

**Figure 2:**
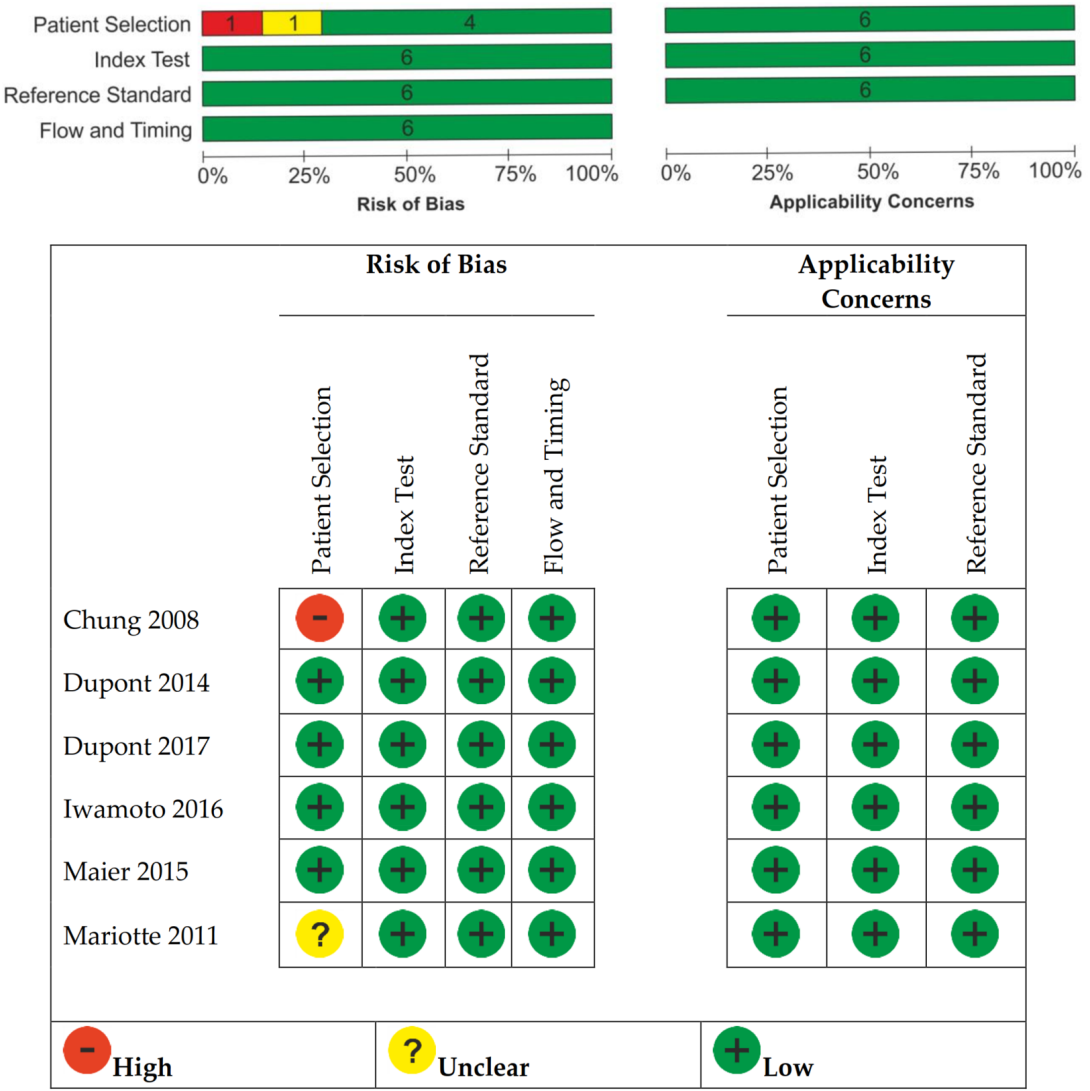
Risk of bias and applicability concerns summary.

The risk of bias is shown in Fig. 2, all studies had no concerns regarding risk of bias or applicability among the four QUADAS-2 domains. We assessed all studies as low risk of bias for this domain. All six included studies collected the index test and reference standard or equivalent as a reference standard at the same time and the flow and timing was appraised as low risk of bias. Our reviewed studies did not recruit predominantly high-risk populations and we assigned low concern in participant selection applicability. We considered Chung et al.^27^ as having high risk of bias in patient selection because all the selected patients from Group 1 were from Tennessee that was demonstrated having high incidence of severe cetuximab hypersensitivity reactions^16^. Risk of bias for patient selection was considered unclear for Mariotte et al.^34^ as it did not report clearly the selection processes. Regarding index test applicability, the studies have enough information to allow us to judge if the index test, its performance, or its interpretations differ from the review aim. Regarding the reference standard, the information was clear; we considered them of low bias.

**Figure 3:**
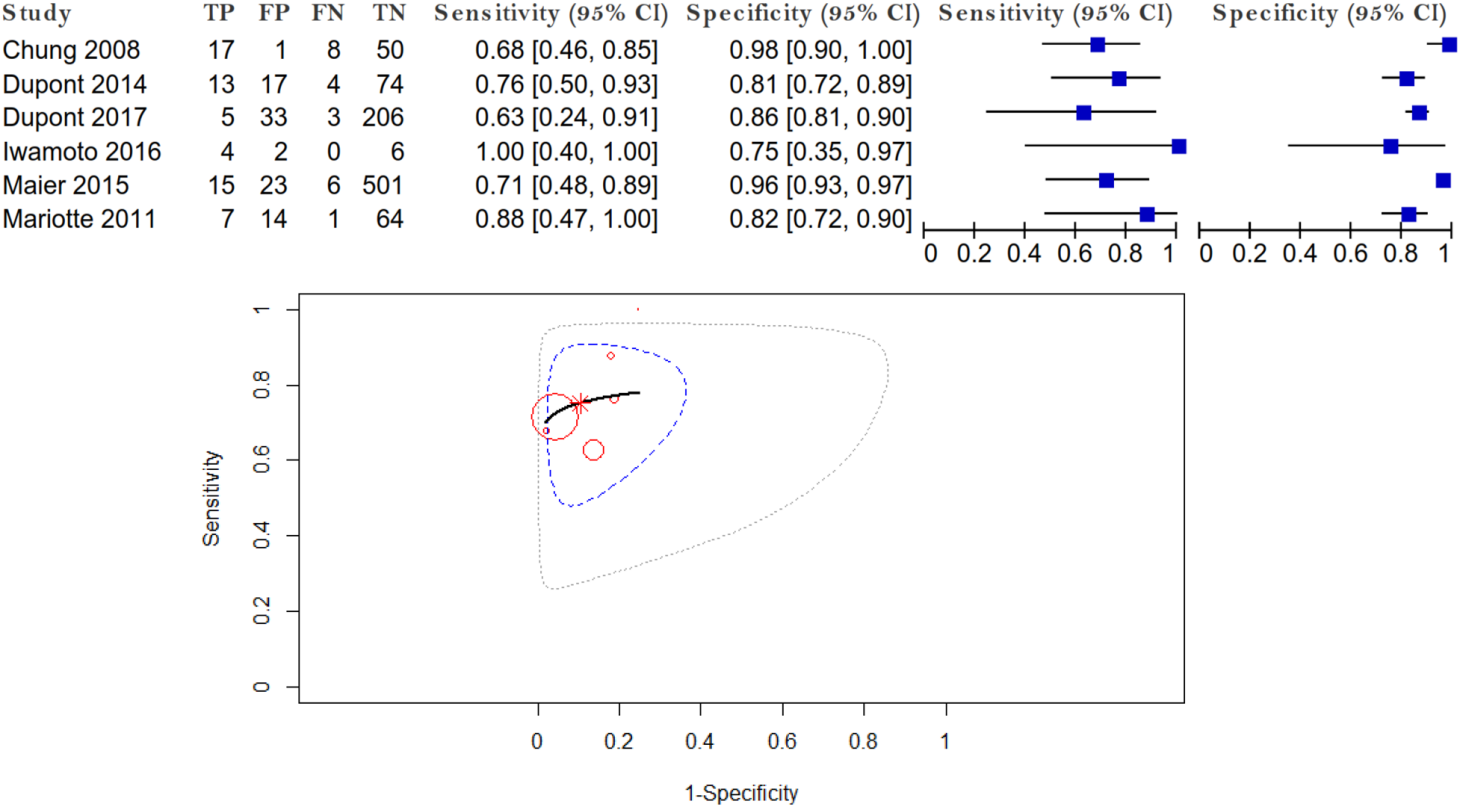
Forest plot and SROC curve.

Forest plot and SROC curve of sensibility and specificity for cetuximab HSR using IgE alpha-gal are given in Fig. 3.

The summary estimate of sensitivity and specificity is shown by the asterisk dot. The pooled sensitivity was 0.725 (95% CI 0.618–0.811). The pooled false positive rate (1-specificity) was 0.118 (95% CI 0.062–0.214). Area under the curve (AUC) was 0.787, representing a good test. The p-value of testing for equality of sensitivities was 0.807, and the p-value of testing for equality of specificities was less than 0.001, providing evidence of no difference in sensitivity and evidence of a difference in specificity. The studies were not heterogeneous: Higgin’s I^2^ = 0%, p = 0.462.

## Discussion

Literature on the use of IgE specific for alpha-gal for diagnosis of cetuximab HSR shows variation in diagnostic accuracy. Sensitivity ranged from 61 to 90%, and specificity ranged from 72 to 97%. The variation in diagnostic accuracy was not wide and we obtained a pooled sensitivity of 73% and a pooled specificity of 88%.

The heterogeneity between studies was not significant; in their study design, they were 5 retrospective and 1 prospective studies^15,22,26,27,29,34^. A heterogeneous element was the criteria used to describe the HSR; some of the studies used the Common Terminology Criteria for Adverse Events Version 4.0 (CTCAE)^15,26,29,35^ while others made their evaluation based on the National Cancer Institute Common Toxicity Criteria^27^, version 3, the classification of Ring and Messmer^34^, and one study provided no data whatsoever about the HSR type^22^.

There were two methods used to assay IgE specific for alpha-gal: 4 studies^15,26,29,34^ measured the cetuximab specific Ig and alpha-gal in serum using in vitro method ELISA—enzyme-linked immunosorbent assay, while two studies^22,27^ measured total and specific IgE using ImmunoCAP, an in vitro test for specific antigen that utilizes a fluoroenzyme immunoassay (FEIA)^35^*.*

Three studies^22,26,29^ reported the association between cetuximab treatment, incidence and severity of systemic hypersensitivity reactions. Approximately 2–5% of the patients treated with cetuximab suffer a HSR, according to the product’s label. This data is supported by Maier et al.^22^ which found an overall incidence of ~ 4%. Such results are consistent with the incidence in the U.S. However, a higher frequency (10.5%) of hypersensitivity reaction was observed in a previous study conducted by Dupont et al.^26^. Grade 1–2 reactions might have been omitted from medical observations, thus, the retrospective collected data might have led to the underestimation of the results. Severe HSRs (grade 3–4) were found in 3.2%^29^ and 4.8%^26^ of patients in the studies populations, thereby corroborating those responses may not be a rare adverse effect.

Grade 3–4 infusion reactions (IRs) happened frequently during the first hour of treatment. In the study population, 15.7% of the patients with grade 1–2 IRs presented recurrent IRs after re-administration of cetuximab, although with no severe symptoms, while another study^26^ shows higher rates (one-third of patients) of developing a subsequent reaction. Treatment with cetuximab was interrupted for grade 3–4 reactions.

The screening for patients at risk of developing a HSR using only clinical criteria appears to be insufficient, since there are conflicting reports regarding the association between HSR and a previous allergic history and also between HSR and cancer localizations, one study showing an increased frequency of reactions in patients treated for head and neck cancer^25^. Six studies^15,22,26,27,29,34^ measured anti-cetuximab IgE in the patients’ serum. Data showed an incidence of 1.4% of severe HSR in the subgroup of patients with low levels of anti-cetuximab IgE and 13.2% in the subgroup with high levels of antibodies, respectively^29^.

Corresponding results were found in two studies^26,34^. Between 71.4 and 76.5% of patients which manifested hypersensitivity reactions were positive for anti-cetuximab IgE. This ratio increased to 87.5% in case of 3–4 grade HSR. Antibodies were found only in 17.9% and 18.7% of those who did not present HSR. Also, three patients from the subgroup who presented grade 2 HSR tested negative for IgE^33^. Another study found that approximately one-third of subjects presenting HSR did not have pre-existing IgE^22^.

Interestingly, the control group and the patients had a similar prevalence of anti-cetuximab IgE and it was higher compared to others, which may be explained by regional variations. No correlation was found between the level of anti-cetuximab IgE in healthy blood donor cohorts or patients and their blood group. The authors found a highly significant odds ratio between IgE + and IgE− patients^34^.

The likelihood of patients positive for anti-cetuximab IgE of experiencing an allergic reaction is 15-times higher than of those lacking IgE^26^. Maier determined that test-positive patients had a 39.5% risk of developing HSR and that levels of IgE > 0.1 kUA/L, the cut-off for a positive value, did not cause reactions in 23 of 38 patients. Conversely, patients negative for antibodies had a high likelihood of not having HSR, statement reinforced by the results of one study^21^ which reported that none of the 37 patients that were alpha-gal tested and obtained negative results experienced anaphylaxis.

Furthermore, most of the severe reactions were associated with IgE antibodies against alpha-gal present in patients before cetuximab treatment^27^. Based on these outcomes, it was speculated that 7 to 8 severe cases of HSR could have been avoided if an alternative treatment had been administered to patients with high pre-treatment levels of anti-cetuximab IgE^29^. These results suggest that evaluation of pre-existing anti-cetuximab IgE might help estimate the risk of HSR^30,31^, although the positive predictive value was determined to be only 0.67, while the negative predictive value was 1.00^15^. As a result, such tests cannot predict a reaction, but they can indicate a higher risk of reaction^31^. In order to confirm sensitization, the authors suggest other conventional diagnostic tests used for allergy, such as basophil activation tests. Because of its complexity and lack standardization, BAT requires an experienced laboratory and a rigorous interpretation. The test is too difficult to be recommended as a screening test, so it is not to be considered as a suitable method that can be used to establish sensitization to cetuximab. Cetuximab-induced basophil activation was observed in a few patients with alpha-gal-specific IgE who were allergic to red meat. However, there have been no studies examining the association between basophil activation tests and the occurrence of cetuximab-induced HSR^15^. There was one case of successful desensitization which allowed the treatment to continue^34^.

With regard to the premedication used to prevent a HSR, one study^15^ supports the use of corticosteroids in limiting the incidence of severe HSR before infusion of cetuximab, and another one^29^ determined that premedication failed to prevent HSR to cetuximab, a fact that can be explained by the mechanism involving pre-existing IgE.

However, the presence of IgE antibodies did not result in HSRs in the majority of patients. Alternatively, the existence of a subgroup of patients who experienced an infusion reaction without pre-existing IgE requires us to consider other pathways by which cetuximab induce allergy^22^.

The lack of positive results of premedication may be explained by the pre-existing IgE in the patients’ serum. The study conducted by Dupont^29^ suggests that corticosteroids and antihistamine administered prior to cetuximab are insufficient in order to successfully avoid HSRs in patients with high concentration of IgE, contrary to previous studies^10,36^. However, administration of antihistamines is still a practice used in many medical centres despite its unproven benefits, thus, calling for an optimization of pre-medication protocols^27^.

The literature describes heterogeneous therapeutic measures in case of low-grade HSR to cetuximab, even re-administration, remote from an anaphylactic reaction, in rare cases^37^. Although in theory resuming treatment is possible after low-grade reactions^38,39^, caution was the common attitude as well as interruption of cetuximab after grade 3 or 4 reactions^26^.

Regarding the regional variations of the IgE antibodies, studies that included patients from different sites across the U.S identified a higher incidence in the Southeast area of the United States^22^. Anti-cetuximab IgE was found in a similar proportion in patients in France^34^. Environmental factors such as tick bites^30,40^ and nematodes^27^, might explain the heterogenicity among different geographic locations^41^. The authors^22^ concluded that in a mobile society it is difficult to correlate the location and the presence of anti-cetuximab IgE or risk of infusion reaction.

From a clinician’s perspective, it is highly important to assess the risk of HSR and to manage the therapy accordingly^42^, especially since preventing severe infusion reaction to cetuximab is challenging both medically and financially. Furthermore, in high-risk areas in the United States cetuximab is replaced in favour of panitumumab, a humanized epidermal growth factor receptor antibody, in colorectal cancer^43^. Unfortunately, the treatment of head and neck cancer offers no such alternative^44^, hence it is important to develop a laboratory test that can identify patients with high anaphylaxis risk. Identifying these patients can lead to the possibility to restore cetuximab as a therapeutic option^21^. To this end, results show that patients negative for IgE antibodies have a high likelihood of not developing HSR^22^. If used, an alpha-gal assay could triage the patients and reduce costs for those with negative results by eliminating the 1:1 monitoring during infusion or the division of cetuximab dose into the test dose and the remaining dose. Conversely, test-positive patients could benefit from carefully monitored conditions while receiving a test dose of cetuximab.

Presently, despite its high negative prospective value, there are not sufficient data to introduce an alpha-gal specific IgE testing as a standard of care^22^. Also, an ELISA test is not able to predict a reaction but rather a higher risk of a reaction, therefore additional tests, such as basophil activation^15^ or skin testing, used for the diagnosis of an allergy could prove helpful in order to establish sensitization to cetuximab^34^.

Our research has some limitations. Three of the studies included in the present review obtained their results from small sample sizes. Furthermore, relevant clinical information may have been missing regarding HSRs due to the studies retrospective nature. A cutoff for IgE was not presented in the included studies, however, sIgE titers above 0.35 IU/ml on ImmunoCAP are correlated strongly with HSR, while values below 0.35 IU/ml on ImmunoCAP or low values on ELISA are much less predictive.

As future research directions, studies could improve the positive prospective value of the laboratory test by including other demographic and establishing a cutoff^21^. From the available data, sIgE detecting methods, mainly ELISA, are available in most countries and they represent commonly used analytical biochemistry assays. Both methods of detection (ELISA and ImmunoCAP) used positive value as a predictive marker. Additionally, ImmunoCAP sIgE to alpha-gal has recently been approved by the FDA for in vitro diagnostic use^45^. This could enable the determination of anti-cetuximab IgE as a predictor for treatment tolerance^26^, similar to how gene mutations, such as KRAS or B-RAF, predict the efficacy of anti-EGFR treatment for colon cancer^46–48^.

In conclusion, despite some discrepancies in the results of the studies included in our systematic review, there is evidence to suggest that the presence of cetuximab specific IgE (anti cetuximab antibody) and/or alpha-gal suggests to increase the risk of developing HSR. The biomarker showed moderate to high accuracy for identifying HSRs related to cetuximab, but special attention should be given in order to avoid or minimize the false positive and false negative results.

Given the high morbidity and mortality of HSRs related to cetuximab, this area of research should be carefully considered by scientists. Thus, more prospective studies are needed to establish a protocol necessary for the proper prediction and avoidance of HSRs related to cetuximab.

## Data Availability

The authors declare that the data in this research is available.
